# Statistical analysis of a cluster-randomized clinical trial on adult general intensive care units in Brazil: TELE-critical care *verSus* usual Care On ICU PErformance (TELESCOPE) trial

**DOI:** 10.5935/0103-507x.20220003-en

**Published:** 2022

**Authors:** Otavio Ranzani, Adriano José Pereira, Maura Cristina dos Santos, Thiago Domingos Corrêa, Leonardo Jose Rolim Ferraz, Eduardo Cordioli, Renata Albaladejo Morbeck, Otávio Berwanger, Lúbia Caus de Morais, Guilherme Schettino, Alexandre Biasi Cavalcanti, Regis Goulart Rosa, Rodrigo Santos Biondi, Jorge Ibrain Figueira Salluh, Luciano César Pontes de Azevedo, Ary Serpa Neto, Danilo Teixeira Noritomi

**Affiliations:** 1 Pulmonary Division, Instituto do Coração, Hospital das Clínicas, Faculdade de Medicina, Universidade de São Paulo - São Paulo (SP), Brazil.; 2 Barcelona Institute for Global Health - Barcelona, Spain.; 3 Department of Critical Care Medicine, Hospital Israelita Albert Einstein - São Paulo (SP), Brazil.; 4 Telemedicine Department, Hospital Israelita Albert Einstein - São Paulo (SP), Brazil.; 5 Postgraduate Program of Health Sciences, Universidade Federal de Lavras - Lavras (MG), Brazil.; 6 Brazilian Research in Intensive Care Network (BRICNet) - São Paulo (SP), Brazil.; 7 Academic Research Organization, Hospital Israelita Albert Einstein - São Paulo (SP), Brazil.; 8 Institute of Social Responsibility, Hospital Israelita Albert Einstein - São Paulo (SP), Brazil.; 9 Research Institute, HCor-Hospital do Coração - São Paulo, (SP), Brazil.; 10 Critical Care Department, Hospital Moinhos de Vento - Porto Alegre (RS), Brazil.; 11 Instituto de Cardiologia do Distrito Federal - Brasília (DF), Brasil.; 12 Instituto D’Or de Pesquisa e Ensino - Rio de Janeiro, (RJ), Brazil.; 13 Emergency Medicine Department, Universidade de São Paulo - São Paulo (SP), Brazil.; 14 Institute for Teaching and Research, Hospital Sírio-Libanês - Sao Paulo, Sao Paulo, (SP), Brazil.; 15 Australian and New Zealand Intensive Care Research Centre, School of Public Health and Preventive Medicine, Monash University - Melbourne, Australia.

**Keywords:** Length of stay, Patient care team, Research design, Data interpretation, statistical, Telemedicine, Hospital mortality, Critical care, Intensive care units, Brazil

## Abstract

**Objective:**

The TELE-critical Care verSus usual Care On ICU PErformance (TELESCOPE) trial aims to assess whether a complex telemedicine intervention in intensive care units, which focuses on daily multidisciplinary rounds performed by remote intensivists, will reduce intensive care unit length of stay compared to usual care.

**Methods:**

The TELESCOPE trial is a national, multicenter, controlled, open label, cluster randomized trial. The study tests the effectiveness of daily multidisciplinary rounds conducted by an intensivist through telemedicine in Brazilian intensive care units. The protocol was approved by the local Research Ethics Committee of the coordinating study center and by the local Research Ethics Committee from each of the 30 intensive care units, following Brazilian legislation. The trial is registered with ClinicalTrials. gov (NCT03920501). The primary outcome is intensive care unit length of stay, which will be analyzed accounting for the baseline period and cluster structure of the data and adjusted by prespecified covariates. Secondary exploratory outcomes included intensive care unit performance classification, in-hospital mortality, incidence of nosocomial infections, ventilator-free days at 28 days, rate of patients receiving oral or enteral feeding, rate of patients under light sedation or alert and calm, and rate of patients under normoxemia.

**Conclusion:**

According to the trial’s best practice, we report our statistical analysis prior to locking the database and beginning analyses. We anticipate that this reporting practice will prevent analysis bias and improve the interpretation of the reported results.

**ClinicalTrials.gov registration:** NCT03920501

## INTRODUCTION

The demand for critical care provided in intensive care units (ICUs) is increasing worldwide.^(1)^ This demand is expected to burden, with high strain and stress, the health systems of low-income and middle-income countries. Lowincome and middle-income countries usually have low availability of ICU beds or experience the effects of significant regional disparities and population aging without adequate control of the countries’ main health determinants.^(2-4)^

One approach to address this increase in demand for care of critically ill patients is improving ICU efficiency, rather than only increasing ICU beds.^(5)^

Daily multidisciplinary rounds (DMRs) are an approach that optimizes ICU care.^(6-8)^ Daily multidisciplinary rounds consist of systematic patient-centered discussions aiming to establish joint therapeutic goals for the following 24 hours of ICU care.^(6)^ Nevertheless, the best method to perform DMR analysis is still lacking. The TELE-Critical Care versus usual Care On ICU PErformance (TELESCOPE) trial aims to evaluate whether an intervention consisting of guided DMRs supported by a remote specialist (intensivist) through telemedicine^(9,10)^ and audit feedback on care performance will reduce ICU length of stay (LOS) compared to a control group.^(11)^

Here, we present the updated and finalized statistical analysis of the TELESCOPE trial. Recruitment for the trial has now been completed, but data collection is ongoing, and no data analysis has been performed.

## METHODS

The TELESCOPE trial is a national, multicenter, controlled, open label, cluster randomized trial. The study tests the effectiveness of DMRs conducted by an intensivist through Telemedicine in Brazilian ICUs. The protocol was approved by the local Research Ethics Committee (IRB) of the coordinating study center (*Hospital Israelita Albert Einstein -* CAAE: 01523118.0.1001.0071) and by the local IRB from each of the 30 ICUs, following Brazilian legislation. The trial is registered with ClinicalTrials.gov (NCT03920501). Further information of the Statistical Analysis Plan (SAP) document can be found on ClinicalTrials.gov.

### Outcomes

The primary, secondary and other exploratory outcomes are described in table 1. The follow-up period to define all outcomes will be truncated at 90 days from ICU admission.

**Table 1:** TELESCOPE trial outcomes

Outcome	Description
Primary outcome	
Patient level	ICU LOS, measured in days, considering the time interval in hours between patients’ ICU admission and ICU discharge times (i.e., transfer to another care facility or another hospital) or ICU death, as defined by the hospital’s system date and time. Date and time will be entered by the health care worker responsible for data collection. ICU LOS will be derived in 24 hours periods with decimal places, as recommended^(12)^
Secondary exploratory outcomes	
Unit level	Classification of the unit according to the profiles defined by the SRU and the SMR.^(13,14)^ The SRU reflects the observed/expected rate of resources used (estimated as ICU length of stay for surviving patients), adjusted by the patient’s severity of illness (SAPS 3).^(15)^ The SMR reflects the observed/expected rate (according to severity score) of hospital deaths. The profiles are a combination of SMR (above or below median) and SRU (above or below median): Each unit can be assigned to one of the four groups: “most efficient” (SMR and SRU < median); “least efficient” (SMR, SRU > median); “overachieving” (low SMR, high SRU), “underachieving” (high SMR, low SRU)^(14)^
Individual level	In-hospital mortality, defined as death by any cause, within the period from the date of ICU admission to the date of hospital discharge or death, whichever comes first Incidence of CLABSI, as defined by the CDC^(16)^ Incidence of VAE, as defined by the CDC^(17)^ Incidence of CAUTI, as defined by the CDC^(18)^ Ventilator-free days at 28 days, defined as the number of days from successfully weaning to Day 28; patients who died before weaning were deemed to have no ventilator-free days Rate of patients receiving oral or enteral feeding, defined as any amount of oral or enteral diet, during ICU stay Rate of patients under light sedation or alert and calm [RASS =-3 to +1] Rate of patients under normoxemia SpO_2_ between 92% and 96%
Other exploratory outcomes	ICU mortality 24-hour ICU readmission rate Proportion of MV use Early reintubation rate (< 48 hours after extubation) Accidental extubation rate Rate of patients with head of bed elevation for patient under MV Rate of CVC use and duration Rate of urinary catheter use and duration Rate of adequate prevention of VTE Rate of patients with adequate glycemic control

ICU - intensive care unit; LOS - length of stay; SRU - standardized resource use; SMR - standardized mortality rate; SAPS 3 - Simplified Acute Physiology Score 3; CLABSI - central line-associated bloodstream infection; CDC - Centers for Disease Control and Prevention; VAE - ventilator-associated event; CAUTI - catheter-associated urinary tract infection; RASS - Richmond Agitation-Sedation Scale; SpO_2_ - peripheral oxygen saturation; MV - mechanical ventilation; CVC - central venous catheter; VTE - venous thromboembolism.

### Eligibility criteria for intensive care units (clusters)

At the cluster level, ICUs of public or philanthropic hospitals, with a minimum of eight ICU beds and on-site registered doctors and nurses, were eligible for inclusion. Only one unit per hospital was allowed.

We excluded ICUs that already presented DMRs, defined as follows:

1) Meetings (DMRs) ≥ 3 times per week, during weekdays, conducted by a certified intensivist and documented in medical records with fixed visit length (> 5 min/patient), used a supporting tool (checklist or standard form), goal-oriented, based on established protocols, including all the patients admitted to the ICU or
2) Monthly management of indicators (audit and feedback) with specific planning. We also excluded specialized ICUs (ICUs exclusively admitting cardiac surgery, neurological, burn patients) and step-down or coronary units.

### Eligibility criteria for patients

At the patient level, all consecutive patients admitted to the ICU, aged 18 years or older after the beginning of the trial, were eligible for inclusion.

We excluded patients admitted to the ICU due to justicerelated issues (since in such circumstances ICU admission or discharge may be determined by law rather than by medical reasons) and patients previously included in the TELESCOPE trial (for the analysis of the primary outcome).

### Intervention

For a description of the intervention, please refer to the TELESCOPE protocol paper.^(11)^ Briefly, the trial intervention consists of DMR led by remote boardcertified intensivists with the local multidisciplinary team (doctor, nurse and physiotherapist). Additionally, ICU performance indicators are presented for each coordinator of the participating ICUs as well as for tele-intensivists, and monthly remote meetings between the local ICU leadership and the respective tele-intensivist are organized to discuss these indicators and to establish potential action plans for improvement. No interventions will be performed in the ICUs randomized to the control group.

### Randomization and masking

After a 2-month observation period (baseline period) in which performance indicators for eligible ICUs were collected without any intervention (with the purpose of obtaining data for randomization, analysis and characterization of the initial ICU status), the ICUs eligible for the study were randomized. The 30 ICUs were then randomly assigned to either the intervention group (n = 15) or the control group (n = 15) using a restricted randomization algorithm that minimizes imbalance between treatment groups across the following baseline covariates at the ICU level:^(19,20)^

1) Number of ICU beds.
2) Mean Simplified Acute Physiology Score 3 (SAPS 3), in points.
3) Mean ICU LOS, in days.
4) Standardized mortality rate (SMR).
5) Standardized resource use (SRU), and
6) A two-category dummy indicator for the Brazilian region where the ICU is located (regions: South and Southeast x regions: North/Northeast/Central-West).

**Figure 1:**
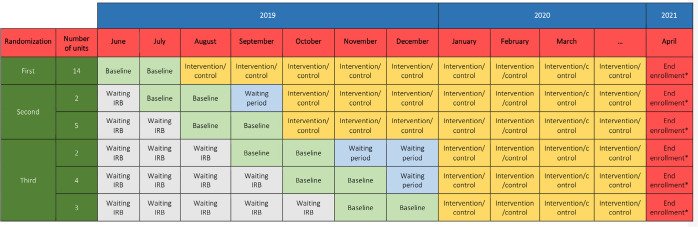
Time periods of 30 units in the TELESCOPE trial. IRB - Research Ethics Committee. * From April, the data collection will continue until hospital outcome or 90-days post-intensive care unit admission. The intervention will be maintained in the whole unit until the last included patient is discharged from the intensive care unit. The waiting period was the period when the intensive care units completed their baseline period of two months and were waiting for more blocks to complete their 2-month period to be randomized as a block.

We followed all the steps recommended by Carter et al. during the application of the minimization algorithm.^(19)^ The randomization was performed three times, including 14 units during the first randomization, followed by seven and nine units (Figure 1). The a priori decision to perform the randomization three times and determine the number of units at each randomization was pragmatic, allowing for ethical approval and completion of the baseline period, respecting the minimum of eight units during the first randomization and a minimum of six for subsequent randomizations.

For the first randomization, we followed the following steps: the database with the baseline data (2 months) was locked; derivation of the six covariates per unit was performed; the algorithm was run, generating the potential combinations of unit allocation, and a random combination of allocations was selected. The order of each unit in the database was randomly sorted before the algorithm, as well as the order of potential allocations. The select allocation was coded as 0 and 1 by the algorithm. To select whether the intervention would be 0 or 1, we performed the final simple randomization, and 0 was allocated to the intervention arm.

For the second and third blocks, we followed the same steps: we entered the baseline data of the previous block with its allocation and the new covariates. The algorithm accounts for the previous block covariates to calculate the new balance between arms. We applied the same random sorting of order and potential unit allocations. The meaning of 0 and 1 was kept the same as the first block randomization (i.e., 0 to intervention and 1 to control) because it must follow the first block ascertainment.^(19)^ To ensure allocation concealment, all units were enrolled prior to randomization and ethics approval, and the ICU and hospital coordinators signed the agreement committing to the trial; the statistician responsible for the randomization list received only the ICU identifier code, unaware of which unit it referred to; the allocation list was sent to the study coordinator, who informed the ICUs about the randomization and allocation.

The allocations were performed after the completion of the baseline on 05 August 2019, 16 October 2019, and 29 January 2020 using the software R (v. 3.5.2).

The intervention is open due to the nature of the study (Tele-ICU rounds, quality improvement meetings and delivery of evidence-based clinical protocols). The steering and scientific committees were blinded to the DMRs and monthly feedback/audit meetings.

### Power calculation

#### Original power calculation

Prior to the start of the trial and baseline period, for the funding application, we estimated that the mean ICU LOS would be 8 (standard deviation - SD - 10) days and an intraclass correlation coefficient (ICC) of 0.018 for general adult public ICUs in Brazil.^(11,21-23)^ Considering a two-arm cluster trial with an ICC of 0.018, for a minimum difference of an average LOS of 1.5 days (8.0 to 6.5 days) and SD of 10 days, power 80%, alpha 5%, we would need a total of 30 clusters (15 intervention units and 15 control units) with an average cluster size of 500 patients per ICU over a period of 18 months. We estimated the coefficient of variation (CV) of cluster size using the expected minimum and maximum method of the cluster size:^(24)^ assuming a minimum cluster size of 350 patients and maximum of 650 patients (i.e., range 300, approximated SD of 75), for a mean size of 500 patients, we would have an approximated CV of 0.15 and maintain 80% power.

#### Power review after the baseline period

We prespecified in the original protocol that once the baseline period was completed for the 30 ICUs, we could review the power calculation. We evaluated the baseline period, and the mean ICU LOS was 7.8 days, SD 9.8 and ICC of 0.087 for a model without covariate adjustment. We had an ICU LOS mean and SD very close to the original power calculation (mean 8 and SD 10) but a higher ICC than predicted. The original power estimation did not account for the use of the data from the baseline period in the model for the primary outcome because we were uncertain if we would have funding to collect individual-level data for the selected ICUs to characterize the baseline period. Therefore, using the framework suggested by Hemming et al.,^(25)^ we re-estimated the power accounting for the baseline period in September 2019. This method uses the cluster autocorrelation (CAC), defined by the ratio of the between-period ICC to the within-period ICC.

Considering a cluster parallel trial with a baseline measure, with a cross-sectional sampling structure, a correlation structure of a two-period decay, and a CV of clusters size of 0.4 (taken from the baseline period), we would maintain 80% power until a CAC value of ≥ 0.906, without considering covariate adjustment. We estimated the CAC on the 20 ICUs used for the estimation of initial ICC, using follow-up periods similar to the TELESCOPE trial, and on all occasions, it was higher than 0.960. Considering the dynamics of ICU LOS and its high correlation over time, we expect the CAC value to be high. Based on this scenario, after a meeting with an external advisory board on 05/10/2020, the steering committee decided to keep the original sample size and power calculation, conditioning it to update the analysis to keep the covariate adjustment and to account for the baseline period.

#### Data collection and management

A detailed description of data collection and management is described in the protocol paper.^(11)^ Data collection procedures will be identical in the ICUs assigned to the control and intervention arms, for the following: at ICU admission; data to ascertain the secondary and tertiary outcomes will be collected daily, including documented treatment goals from the DMR; upon ICU discharge; and at hospital discharge, date and time and outcome.

### Statistical methods analysis

#### General analysis principles

##### Analysis population

Primary statistical analyses will be performed according to the intention-to-treat principle. Patient outcomes will be analyzed according to the randomization of the ICU in which each patient was in, regardless of whether the intervention was applied in that ICU. The baseline period is defined as the first two months of data collection in each ICU. The period for the evaluation of the intervention will be defined as the day after randomization; thus, patients admitted to the ICUs the next day after randomization will be counted as part of the intervention/ control period. Primary statistical analysis will also consider the baseline period in the analyses, while patients admitted to some of the ICUs during the “Waiting period” (between baseline and randomization) will be excluded.

##### Database locking

All analyses planned in this statistical analysis will be conducted only after database locking. The data management and checks for missing data and consistency will be conducted blinded to the ICU code and allocation.

##### Missing data

We will perform multiple imputation if the missing data on the core variables are > 5% under the assumption that the pattern of missing data is missing at random (MAR), conditional on the observed data.^(26)^ Core variables are defined as the covariates to be used in the main analysis of the primary outcome: SAPS-3 score, type of ICU admission, invasive mechanical ventilation at ICU admission, number of ICU beds at baseline, region of Brazil where the ICU is located, ICU performance at baseline and order of randomization. We will follow the recommended standard steps of multiple imputation.^(26)^ We will include the outcome and account for the clustered structure of the data. The imputation model will have the covariates used in the main model and auxiliary variables. We will start with 20 imputed datasets and change the number of imputed datasets based on the fraction of missing information (FMI).^(27)^ We will pool the estimates using Rubin’s rules. The random number seed will be set to 2605.

For the severity scores, SAPS 3 and SOFA, we will consider “zero points” or “normal values” where data are missing. If we perform multiple imputation, we will impute the final composite score.

##### Multiplicity

Prespecified secondary outcomes and subgroup analyses will not be adjusted for multiple comparisons. They should, therefore, be interpreted as exploratory.

##### Other issues

The TELESCOPE trial has continued during the COVID-19 pandemic. It is likely that the pandemic changed the usual characteristics of the admitted patients, such as if an ICU from the TELESCOPE trial became a reference for COVID-19 patients and non-COVID-19 patients. The ICU performance, both at the control and intervention arms, could have influenced the decision-makers at the federal, state and municipal levels to decide whether to designate an ICU for COVID-19. As ICU performance is, in this case, postrandomization, we will not adjust for whether an ICU is a reference center for COVID-19 in the analysis; otherwise, it will break the advantages of randomization.

Neither the steering committee or tele-intensivists was responsible for deciding whether an ICU would be a reference center for COVID-19.

#### Statistical analyses

We will follow the framework proposed in the literature about optimizing power and properly accounting for time in cluster parallel randomized trials with a baseline period (longitudinal cross-sectional cluster trials).^(25,28,29)^ Thus, we will consider the baseline individual data allowing the secular trend to randomly vary across clusters by extending the random-effects components with interactions between cluster and time period (baseline *versus* after randomization).^(28)^ This period also allows for the ICC to differ from observations that are made in the same or different time periods and for estimating two ICCs (the “within-period ICC” and the “between-period ICC”) used to estimate the CAC (the ratio of the between-period and within-period ICCs).

#### Analysis of the primary outcome

The linear mixed model for the primary outcome will be as in Equation 1.

Equation (1) $$Y_{ijk}=\beta_{0}+\beta_{1}X_{ij}+\beta_{2j}+\beta_{3}Z_{ijk}+\beta_{4}L_{ij}+{µ}_{i}+v_{ij}+\varepsilon_{ijk}$$

for Cluster *i*, time period *j* and Participant *k*, where X_*i*_ denotes the trial arm for Cluster *i* (coded 0 or 1), and *j* denotes the time period in which Participant *k* in Cluster *i* is assessed (0 for baseline, 1 for after randomization). The individual error term is assumed to be Normally distributed and independent of n_*ij*_ and m_*i*_. Random terms are assumed to be Normally distributed, and n_*ij*_ is assumed independent of m_*i*_. Z_*ijk*_ and L_*ij*_ are the matrix of covariates at the individual level and unit level, respectively. b_*1*_ is the coefficient of interest for the trial, i.e., the estimate for the difference between those receiving the intervention and those in the control group.

For the primary outcome, we will include the index admission of a patient, i.e., we will not include ICU readmissions. The primary outcome - ICU LOS - will be log-transformed to account for the normality of the residuals of the linear mixed model. These two steps (not including readmissions and log-transformation) were used for the power calculations.

We will use the identity link in the model for the primary outcome and Satterthwaite’s degree-of-freedom correction.^(20,30,31)^

#### Per-protocol analysis for the primary outcome

We planned a substudy to evaluate a per-protocol analysis in the TELESCOPE trial. We will use the principles of causal inference and deal with postrandomization confounding and biases, keeping the inference based on randomization.^(32)^ We plan to use the principal stratification approach.^(33)^

#### Sensitivity analyses for the primary outcome

These two sensitivity analyses regarding the primary outcome were prespecified in the original protocol.

#### Readiness to discharge

The primary outcome - ICU LOS - is an outcome that also depends on factors outside of the ICU for those who improved during the ICU stay, such as ward bed availability.^(34)^ For this reason, we specified to also measure the ICU LOS in terms of readiness to discharge, i.e., days from ICU admission to the first day the attending team determined that the patient was ready to be discharged alive. This variable was measured during the daily data collection and will be measured as counts because we will not have the appropriate amount of time for its measurement. The attending clinicians were not aware that this would be an outcome of the TELESCOPE trial. We will fit Equation 1 using generalized linear mixed models to accommodate it using a Poisson or negative binomial family with a log link.

#### Competing risk of death

The primary outcome - ICU LOS - is subject to the competing risk of death.^(12)^ Therefore, the ICU LOS represents a composite summary of two processes: time to ICU discharge alive and time to ICU death. There are alternatives to address this scenario, such as fitting competing risk models, modeling time to discharge alive and considering time to ICU death as a competing event, analyzing LOS separately between ICU survivors and nonsurvivors, and statistically weighting the LOS for those who died, among others. As expected, the potential difference in the results of the primary outcome analysis are likely attributable to determining whether the intervention has an effect on mortality. We consider this a secondary analysis because, a priori, we did not expect a major impact of the intervention on mortality. In this sensitivity analysis, we will use the competing risk framework, presenting the analysis with cause-specific hazard ratios and subdistribution hazard ratios for the time to discharge alive.^(35)^ The models will be adjusted for the same six covariates as the primary analysis, and the baseline period will be accounted for as the mean ICU LOS at the unit level in the baseline period. We will account for the correlated data structure with a “shared frailty model”.

#### Analysis of secondary outcomes at the intensive care unit level

The ICU performance classification will be defined in the baseline and after the intervention periods without considering if the unit is in the intervention or control group. Thus, we will estimate if there will be a shift toward better performance for the ICUs in the intervention group, i.e., if there will be more commonly “most efficient” and “overachieving” ICUs in the intervention group. Based on the background that the intervention might be more efficient over time, in an exploratory analysis, we will analyze the ICU performance classification at the last 3 months of the intervention. For the ICU-level outcomes, we will include all patients who met all the inclusion criteria, and we will include readmissions.

#### Analyses of secondary outcomes at the patient level

For all secondary outcomes at the individual level, we will fit Equation 1 using generalized linear mixed models to accommodate each secondary outcome (e.g., logistic mixed model for mortality; Poisson/negative binomial for rates, etc.). We will adjust for the same covariates, except when it is not possible because of the outcome type. For instance, ventilator-associated events can be measured only in patients with invasive mechanical ventilation.

For the secondary outcomes that involve rates or patientdays, catheter-days, etc., we will include all patients who met the inclusion criteria, and we will include readmissions.

#### Covariate adjustment

We prespecified that the analyses for the primary and secondary outcomes would be adjusted by covariates in the original protocol. Based on the literature on the determinants of ICU LOS, we will adjust by three patientlevel covariates and four ICU-level covariates:

- SAPS-3 (continuous term).
- Type of ICU admission (three categories: medical, elective surgical, unplanned surgical).
- Invasive mechanical ventilation at ICU admission (two categories: yes, no).
- Number of ICU beds at baseline (continuous term).
- Brazilian region where the ICU is located (two categories: South/Southeast, North/Northeast/Central-West).
- ICU performance at baseline (four categories: most efficient, least efficient, overachieving, underachieving).
- Groups of randomization (three categories: first, second and third blocks).

Subgroup analysis for the primary outcome will be conducted for six subgroups as defined below:

- Type of ICU admission (three categories: medical, elective surgical, unplanned surgical).
- Tertiles of SAPS 3 score (defined by “ntile(SAPS3.3)”).
- Invasive mechanical ventilation at ICU admission (two categories: yes, receive invasive mechanical ventilation at ICU admission; no, receive other respiratory support rather than invasive mechanical ventilation or none).
- Age groups (three categories: 18 - 39 years, 40 - 59 years, 60+ years).
- ICU performance at baseline (four categories: most efficient, least efficient, overachieving, underachieving).
- Calendar time from the intervention in trimesters (as categorical).

Subgroups will be analyzed by adding an interaction term between the *X_ij_* in Equation 1 and the subgroup of interest and a term for the fixed effect of the subgroup of interest if it is not already a covariate. The p value for the interaction will be evaluated by a likelihood ratio test comparing the model without the interaction and with the interaction.

#### Reporting

We will follow the CONSORT extension for clusterrandomized trials.^(36)^ The results of the TELESCOPE trial will be reported transparently, regardless of its results, and disseminated to the participating centers, funding agency, scientific community, and community. Any deviation from the protocol and this SAP will be highlighted.

## References

[1] Adhikari NK, Fowler RA, Bhagwanjee S, Rubenfeld GD (2010). Critical care and the global burden of critical illness in adults. Lancet.

[2] Ranzani OT, Bastos LS, Gelli JG, Marchesi JF, Baião F, Hamacher S (2021). Characterisation of the first 250,000 hospital admissions for COVID-19 in Brazil: a retrospective analysis of nationwide data. Lancet Respir Med.

[3] Veras RP, Oliveira M (2018). Aging in Brazil: the building of a healthcare model. Cienc Saude Colet.

[4] GBD 2016 Brazil Collaborators (2018). Burden of disease in Brazil, 1990-2016: a systematic subnational analysis for the Global Burden of Disease Study 2016. Lancet.

[5] Valley TS, Noritomi DT (2020). ICU beds: less is more? Yes. Intensive Care Med.

[6] Lane D, Ferri M, Lemaire J, McLaughlin K, Stelfox HT (2013). A systematic review of evidence-informed practices for patient care rounds in the ICU. Crit Care Med.

[7] Kim MM, Barnato AE, Angus DC, Fleisher LA, Fleisher LF, Kahn JM (2010). The effect of multidisciplinary care teams on intensive care unit mortality. Arch Intern Med.

[8] Pronovost PJ, Angus DC, Dorman T, Robinson KA, Dremsizov TT, Young TL (2002). Physician staffing patterns and clinical outcomes in critically ill patients: a systematic review. JAMA.

[9] Wilcox ME, Adhikari NK (2012). The effect of telemedicine in critically ill patients: systematic review and meta-analysis. Crit Care.

[10] Young LB, Chan PS, Lu X, Nallamothu BK, Sasson C, Cram PM (2011). Impact of telemedicine intensive care unit coverage on patient outcomes: a systematic review and meta-analysis. Arch Intern Med.

[11] Noritomi DT, Ranzani OT, Ferraz LJ, Dos Santos MC, Cordioli E, Albaladejo R, Serpa Neto A, Correa TD, Berwanger O, de Morais LC, Schettino G, Cavalcanti AB, Rosa RG, Biondi RS, Salluh JI, Azevedo LC, Pereira AJ, TELESCOPE Trial Investigators (2021). TELE-critical Care verSus usual Care On ICU PErformance (TELESCOPE): protocol for a cluster-randomised clinical trial on adult general ICUs in Brazil. BMJ Open.

[12] Harhay MO, Ratcliffe SJ, Small DS, Suttner LH, Crowther MJ, Halpern SD (2019). Measuring and analyzing length of stay in critical care trials. Med Care.

[13] Rapoport J, Teres D, Lemeshow S, Gehlbach S (1994). A method for assessing the clinical performance and cost-effectiveness of intensive care units: a multicenter inception cohort study. Crit Care Med.

[14] Rothen HU, Stricker K, Einfalt J, Bauer P, Metnitz PG, Moreno RP (2007). Variability in outcome and resource use in intensive care units. Intensive Care Med.

[15] Moreno RP, Metnitz PG, Almeida E, Jordan B, Bauer P, Campos RA, Iapichino G, Edbrooke D, Capuzzo M, Le Gall JR, SAPS 3 Investigators (2005). SAPS 3--From evaluation of the patient to evaluation of the intensive care unit. Part 2: Development of a prognostic model for hospital mortality at ICU admission. Intensive Care Med.

[16] Centers for Disease Control and Prevention (CDC), National Healthcare Safety Network (NHSN) (2018). Bloodstream Infection Event (Central LineAssociated Bloodstream Infection and Non-central Line Associated Bloodstream Infection).

[17] Centers for Disease Control and Prevention (CDC), National Healthcare Safety Network (NHSN) (2018). Pneumonia (Ventilator-associated [VAP] and non-ventilator-associated Pneumonia [PNEU]) Event.

[18] Centers for Disease Control and Prevention (CDC), National Healthcare Safety Network (NHSN) (2018). Urinary Tract Infection (Catheter-Associated Urinary Tract Infection [CAUTI] and Non-Catheter-Associated Urinary Tract Infection [UTI]) and Other Urinary System Infection [USI]) Events.

[19] Carter BR, Hood K (2008). Balance algorithm for cluster randomized trials. BMC Med Res Methodol.

[20] Richards-Belle A, Mouncey PR, Wade D, Brewin CR, Emerson LM, Grieve R, Harrison DA, Harvey S, Howell D, Mythen M, Sadique Z, Smyth D, Weinman J, Welch J, Rowan KM, POPPI Trial Investigators (2018). Psychological Outcomes following a nurse-led Preventative Psychological Intervention for critically ill patients (POPPI): protocol for a cluster-randomised clinical trial of a complex intervention. BMJ Open.

[21] Associação de Medicina Intensiva Brasileira (AMIB) (2018). Brazilian ICUs Project. Profile of the ICUs.

[22] Soares M, Bozza FA, Angus DC, Japiassú AM, Viana WN, Costa R (2015). Organizational characteristics, outcomes, and resource use in 78 Brazilian intensive care units: the ORCHESTRA study. Intensive Care Med.

[23] Ranzani OT, Simpson ES, Augusto TB, Cappi SB, Noritomi DT, AMIL Critical Care Group (2014). Evaluation of a minimal sedation protocol using ICU sedative consumption as a monitoring tool: a quality improvement multicenter project. Crit Care.

[24] Eldridge SM, Ashby D, Kerry S (2006). Sample size for cluster randomized trials: effect of coefficient of variation of cluster size and analysis method. Int J Epidemiol.

[25] Hemming K, Kasza J, Hooper R, Forbes A, Taljaard M (2020). A tutorial on sample size calculation for multiple-period cluster randomized parallel, cross-over and stepped-wedge trials using the Shiny CRT Calculator. Int J Epidemiol.

[26] Sterne JA, White IR, Carlin JB, Spratt M, Royston P, Kenward MG (2009). Multiple imputation for missing data in epidemiological and clinical research: potential and pitfalls. BMJ.

[27] Madley-Dowd P, Hughes R, Tilling K, Heron J (2019). The proportion of missing data should not be used to guide decisions on multiple imputation. J Clin Epidemiol.

[28] Hemming K, Taljaard M, Forbes A (2017). Analysis of cluster randomised stepped wedge trials with repeated cross-sectional samples. Trials.

[29] Copas AJ, Hooper R (2020). Cluster randomised trials with different numbers of measurements at baseline and endline: sample size and optimal allocation. Clin Trials.

[30] Hussey MA, Hughes JP (2007). Design and analysis of stepped wedge cluster randomized trials. Contemp Clin Trials.

[31] Leyrat C, Morgan KE, Leurent B, Kahan BC (2018). Cluster randomized trials with a small number of clusters: which analyses should be used?. Int J Epidemiol.

[32] Hernán MA, Robins JM (2017). Per-protocol analyses of pragmatic trials. N Engl J Med.

[33] Agbla SC, De Stavola B, DiazOrdaz K (2020). Estimating cluster-level local average treatment effects in cluster randomised trials with non-adherence. Stat Methods Med Res.

[34] Harhay MO, Ratcliffe SJ, Halpern SD (2017). Measurement error due to patient flow in estimates of intensive care unit length of stay. Am J Epidemiol.

[35] Wolkewitz M, Cooper BS, Bonten MJ, Barnett AG, Schumacher M (2014). Interpreting and comparing risks in the presence of competing events. BMJ.

[36] Campbell MK, Piaggio G, Elbourne DR, Altman DG, CONSORT Group (2012). Consort 2010 statement: extension to cluster randomised trials. BMJ.

